# Novel Nonphosphorylated Peptides with Conserved Sequences Selectively Bind to Grb7 SH2 Domain with Affinity Comparable to Its Phosphorylated Ligand

**DOI:** 10.1371/journal.pone.0029902

**Published:** 2012-01-11

**Authors:** Dan Zhang, Chen Shao, Siqi Hu, Sucan Ma, Youhe Gao

**Affiliations:** 1 National Key Laboratory of Medical Molecular Biology, Department of Physiology and Pathophysiology, Institute of Basic Medical Sciences, Chinese Academy of Medical Sciences/School of Basic Medicine, Peking Union Medical College, Beijing, China; 2 State Key Laboratory for Molecular Virology and Genetic Engineering, Institute of Pathogen Biology, Chinese Academy of Medical Sciences, Peking Union Medical College, Beijing, China; Bioinformatics Institute, Singapore

## Abstract

The Grb7 (growth factor receptor-bound 7) protein, a member of the Grb7 protein family, is found to be highly expressed in such metastatic tumors as breast cancer, esophageal cancer, liver cancer, etc. The src-homology 2 (SH2) domain in the C-terminus is reported to be mainly involved in Grb7 signaling pathways. Using the random peptide library, we identified a series of Grb7 SH2 domain-binding nonphosphorylated peptides in the yeast two-hybrid system. These peptides have a conserved GIPT/K/N sequence at the N-terminus and G/WD/IP at the C-terminus, and the region between the N-and C-terminus contains fifteen amino acids enriched with serines, threonines and prolines. The association between the nonphosphorylated peptides and the Grb7 SH2 domain occurred *in vitro* and *ex vivo*. When competing for binding to the Grb7 SH2 domain in a complex, one synthesized nonphosphorylated ligand, containing the twenty-two amino acid-motif sequence, showed at least comparable affinity to the phosphorylated ligand of ErbB3 *in vitro*, and its overexpression inhibited the proliferation of SK-BR-3 cells. Such nonphosphorylated peptides may be useful for rational design of drugs targeted against cancers that express high levels of Grb7 protein.

## Introduction

Grb7 protein is associated with amplification and invasion of many solid cancers, including those of the breast [1], esophagus [2], pancreas [3] and lymphocytic leukemia [4]. Via the SH2 domain, Grb7 protein participates in many signaling pathways, such as those associated with insulin [5], ErbB2, ErbB3 and ErbB4 [6], FAK [7], c-Kit/SCFR [8] and FGFR [9].

The SH2 domain plays vital roles in signaling pathways by acting as adaptors, kinases or scaffolds [10]. The SH2 domain contains a central anti-parallel β sheet surrounded by two α helices [11]. It has a positively charged binding cavity [12] that promotes its association with phosphotyrosine motifs [13]. Although different SH2 domains prefer distinct phosphorylated motifs [14], certain SH2 domains are more flexible in their motif preference [15].

SH2 domains also associate with phosphotyrosine-independent motifs. For instance, the T/S-x-x-x-x-V/I (x represents any amino acid) motif is recognized by the SAP/SH2D1A SH2 domain [16], phosphorylated serines and threonines residues are identified by the SH2 domain of ABL [17], nonphosphorylated peptides are distinguished by the SH2 domains of Grb2 and Grb7 [18], [19], the nonphosphorylated SIY(442)DNV motif is able to associate with the cten (C-terminal tensin like) SH2 domain, and the serine and tyrosine residues in this motif are necessary for the interaction, further more, a phosphorylation modification on the tyrosine residue slightly attenuates its affinity to the cten SH2 domain [20].

The Grb7 protein family, identified through cDNA expression libraries encoding phosphotyrosine receptor targets [21], [22], is an SH2 domain protein clan that recruits downstream molecules and participates in important cellular signaling pathways [23]. Three members of this family are Grb7, Grb10 and Grb14. Grb7 participates in cell migration and angiogenesis, Grb10 is involved in cell metabolic control and development amplification and Grb14 has roles in cell metabolic regulation and proliferation [23]. Differences exist between the SH2 domain of Grb7 family proteins and the SH2 domain of other conventional SH2 proteins. The SH2 domain of Grb7 family members displays a more permissive structure by having three more residues in the DE loop and five less residues in the CD loop [24]. SH2 domains of Grb10γ and Grb7 form dimers, whereas other SH2 domains are normally monosomatic [25]. There are also dissimilarities in the SH2 domains of the three Grb7 family members. Grb10 and Grb14 have the most similar SH2 domain sequences [21], the SH2 domain of Grb10 shares approximately 90% similarity with that of Grb14. The SH2 domain of Grb7, but not that of Grb10 and Grb14, associates with β-turn peptides, which is similar to the SH2 domain of Grb2 [26].

Many drugs are designed to target the SH2 domain of Grb7. Krag and colleagues have identified a nonphosphorylated peptide, designated the G7-18NATE, that binds to the SH2 domain of Grb7. The dissociation constant (K_d_) of the G7-18NATE peptide for binding to the Grb7 SH2 domain is approximately 13.2 µM, and that of the ErbB3 pY(1180) ligand is approximately 1.15 µM [27]. The G7-18NATE peptide effectively inhibits the association between Grb7 and ErbB3 [28], pancreatic cancer migration [3] and breast cancer cell proliferation [29]. This peptide also serves as a model for chemical drug design targeting the Grb7 protein [30].

## Results

### Novel nonphosphorylated twenty-two amino residue peptides harboring conserved sequences at both ends selectively bind to the Grb7 SH2 domain

The Grb7 SH2 domain was used to screen the random peptide library in the yeast two-hybrid system, and nine positive clones were identified (Table S1). The twenty-two amino acid sequence containing GIPT/K/N at the N-terminus and G/W/D/I/P at the C-terminus was the most notable feature of the positive clones (Table 1). Although the Grb2 SH2 domain was reported to recognize nonphosphorylated peptide [18], we did not find any positive clone binding to the Grb2 SH2 domain in our system, neither to the ABL_1 SH2 domain.

**Table 1:** Nonphosphorylated peptides interacting with the Grb7 SH2 domain (abridged sequences of conserved amino acid sequences).

No. of peptide	Sequences
3 and 52^a^	GIPT *HSSPQYSPPSTYSPP* GDP
10	GIPN *YTPTTPTLLLTRPLP* GIP
16	GIPT *ATTSPYENANPPHQT* WDP
41-A^b^41-B	GIPT *QPTTSSEPSPPSNPP* WDPGIPT *HHQNDTYNSPHAHPN* RDP
60	RNSY *FTFLPARSLYLIKTH* WDP
67	GIPK *AQNTTATPEQHASPT* GIP
98	GIPN *QDPPAATQSPSQETT* WDP
106	GIPT *STPNTHSTTSHHKNP* WDP

Notes:

a) No. 3 and No. 52 were the same clones.

b) There were two twenty-two residue motifs in the peptide No. 41, with sixteen residues between them, designated 41-A and 41-B, respectively. The full nonphosphorylated peptide sequences were accessible in Table S1.

To see whether the conserved residues were required for association with the Grb7 SH2 domain, the peptide 10 was used as the mutation template to design three mutated peptides: the N-GIPT deletion mutation (10AM), the C-GIP deletion mutation (10BM) and the both N-GIPT and C-GIP deletion mutation (10CM). None of the three mutations were recognized by the Grb7 SH2 domain in the yeast two-hybrid system. This indicates that the peptide requires the conserved residues at each end to recognize the Grb7 SH2 domain.

The SH2 domain of Grb14, which is another member of the Grb7 protein family, was also used to screen the random peptide library with yeast two-hybrid analysis. However, no positive clone was identified. The nine clones (Table 1) that bound to the Grb7 SH2 domain also did not interact with the Grb14 SH2 domain.

### One nonphosphorylated peptide and the Grb7 SH2 domain are co-immunoprecipitated from transfected HEK293Tcells

**Figure 1:**
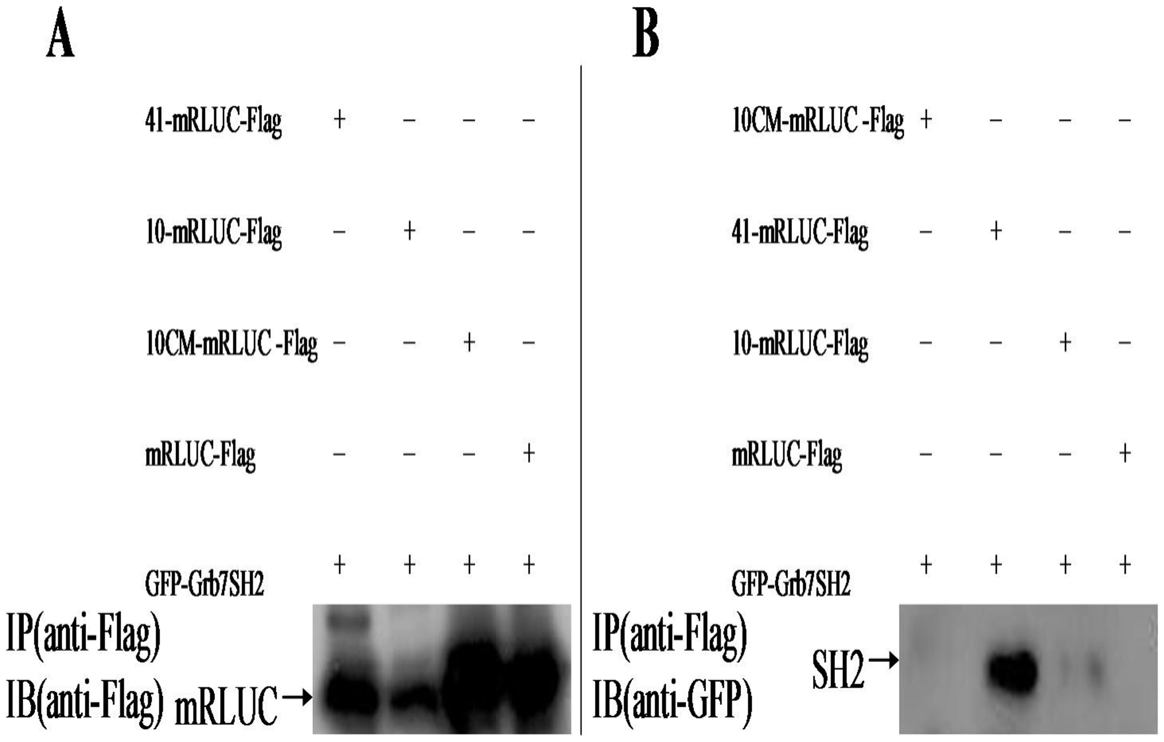
The nonphosphorylated peptide 41 and the Grb7 SH2 domain are co-immunoprecipitated from transfected HEK293T cells. **A**) Expression of nonphosphorylated peptides. **B**) Interactions between nonphosphorylated peptides and the Grb7 SH2 domain.

The 10, 41 and 10CM sequences were fused to the mRLUC-3×Flag and co-transfected with the EGFP-Grb7 SH2 into HEK293T cells using calcium phosphate method respectively. Fusion protein expression was first verified. The expression of peptide 10 was low (Figure 1A). In co-immunoprecipitation assays, peptide 41, but not the peptide 10CM or the control, pulled down the Grb7 SH2 domain (Figure 1B).

### The binding of the Grb7 SH2 domain to the nonphosphorylated peptides is detected by Bioluminescence Resonance Energy Transfer (BRET)

**Figure 2:**
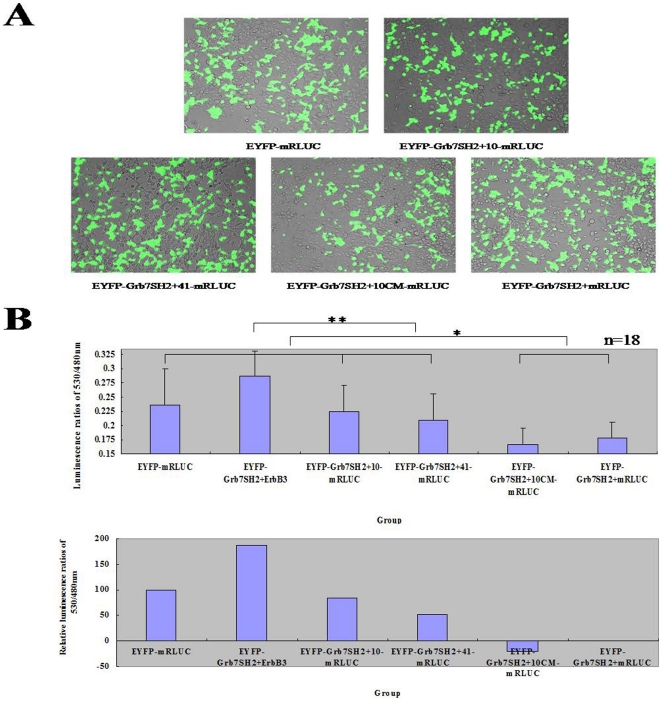
BRET assay of interactions between nonphosphorylated peptides and the Grb7 SH2 domain. **A**) Overlaid images of transfected EYFP-fused proteins in living HEK293T cells at 480 nm. **B**) Quantification of the BRET luminescence ratios. The direct luminescence ratios of 530/480 nm were on the top, and the relative luminescence ratios of 530/480 nm were on the bottom. The BRET reaction was initiated by the coelenterazine (5.9 µM). The statistical analysis was based on eighteen group ratios, * represented statistical significance with p<0.05 and ** represented statistical significance with p<0.01.

The EYFP-tagged Grb7 SH2 domain was co-transfected with the mRLUC-tagged nonphosphorylated peptides into HEK293T cells using calcium phosphate (Figure 2A). The EYFP-mRLUC plasmid was transfected as positive control. The luminescence ratios of 530/480 nm for the EYFP-mRLUC positive control, the interacting EYFP-tagged Grb7 SH2 domain and mRLUC-tagged peptide 10 and 41 groups, were significantly stronger than the luminescence produced by the non-interacting EYFP-tagged Grb7 SH2 domain and the mRLUC-tagged peptide 10CM or control vector groups (Figure 2B). The strongest luminescence was produced by the EYFP-tagged Grb7 SH2 domain and mRLUC-tagged ErbB3 group.

### The nonphosphorylated twenty-two amino acid-motif ligand has at least comparable affinity to the phosphorylated ligand for the Grb7 SH2 domain *in vitro*

**Figure 3:**
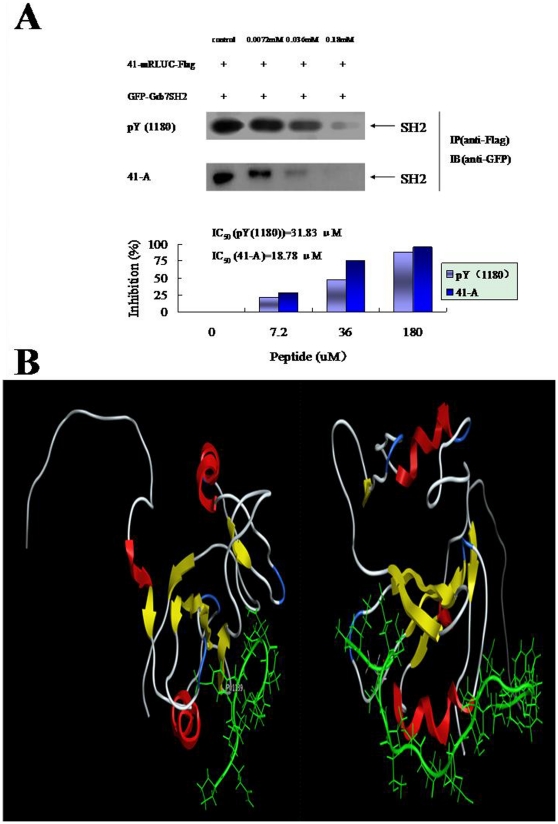
The nonphosphorylated peptide has at least comparable binding affinity to the phosphorylated ligand pY (1180) for the Grb7 SH2 domain. **A**) Different concentrations of synthesized phosphorylated ligands pY (1180) (DEEYEpY(1180)MNRRR) from ErbB3 and the nonphosphorylated ligands 41-A (VAVGIPTQPTTSSEPSPPSNPPWDPGRV) from the nonphosphorylated ligand 41 were added respectively into the interacting complexes of Grb7 SH2 domain and peptide 41 (on the top). The bands were scanned and IC_50_ values were calculated (on the bottom). **B**) Structural modeling for the nonphosphorylated peptide 41-A (VAV GIPTQPTTSSEPSPPSNPPWDPGRV) binding to the Grb7 SH2 domain was performed with MOE. Left was the structural model of the Grb7 SH2/pY (1139) of ErbB2 [31], right was that of the Grb7 SH2/41-A. Red section represented α helix; yellow represented β strand; white represented loop in the Grb7 SH2 domain; green represented the binding ligand; for the nonphosphorylated peptide, left was the N-terminus, and right was the C-terminus.

To see whether the nonphosphorylated peptide could effectively compete with phosphorylated ligand for binding to the Grb7 SH2 domain, the phosphorylated ligand pY (1180) (DEEYEpY(1180)MNRRR), which was previously shown to interact with the Grb7 SH2 domain [6], and the nonphosphorylated 41-A ligand (VAVGIPTQPTTSSEPSPPSNPPWDPGRV), which contains the first twenty-two amino acid-motif close to the N-terminus of peptide 41, were examine for their abilities to interfere with the GFP-Grb7 SH2 domain and 41-mRLUC-3×Flag complex using immunoprecipitation with anti-GFP antibody. As shown in Figure 3A, the nonphosphorylated peptide 41 could bind to the Grb7 SH2 domain in the absence of peptide pY (1180) and peptide 41-A. Low concentrations of peptide pY (1180) and peptide 41-A (0.0072 mM and 0.036 mM) did not significantly interfere with binding of the Grb7 SH2 domain to peptide 41. However, concentration of peptide pY (1180) and peptide 41-A reached 0.18 mM, the interaction between the Grb7 SH2 domain and peptide 41 was nearly abolished. The disruptive effect of peptide 41-A was even stronger than that of the pY (1180) ligand, and phosphorylated peptide pY (1180) (IC_50_ = 31.83 µM) and nonphosphorylated peptide 41-A (IC_50_ = 18.78 µM) had at least comparable binding affinities to the Grb7 SH2 domain.

Because the peptide pY (1180) abolished the association between the Grb7 SH2 domain and nonphosphorylated peptide 41 (Figure 3A), we speculated that the binding interface of the Grb7 SH2 domain for the nonphosphorylated twenty-two amino acid-motif might be the same for the pY(1180) ligand. Structural modeling based on the Molecular Operating Environment (MOE) was performed to illustrate the possible binding site (Figure 3B, right), and the Grb7 SH2/pY (1139) ligand of ErbB2 was used as control (Figure 3B, left) [31]. In this model, the binding interface on the SH2 domain with a known structure was set as rigid and the ligand was set as flexible.

### Nonphosphorylated peptide inhibits the proliferation of SK-BR-3 breast cancer cells

**Figure 4:**
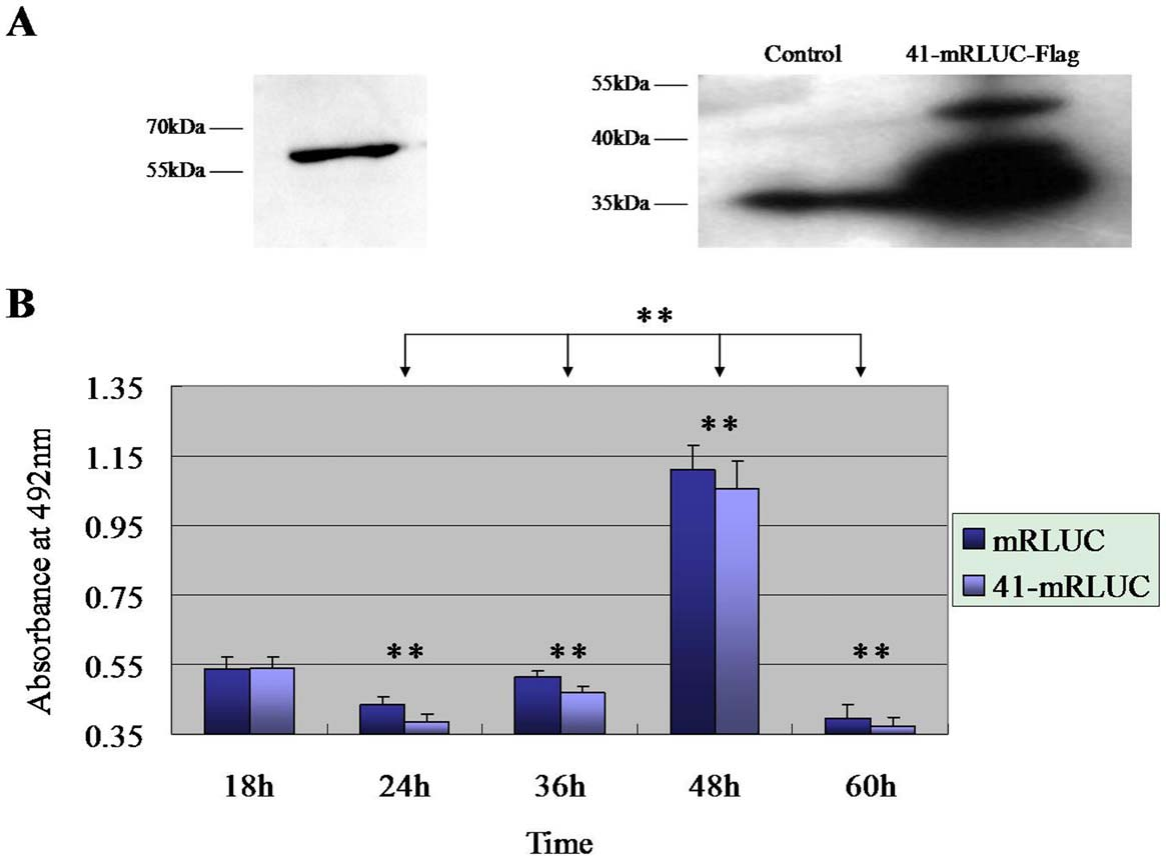
Effect of the nonphosphorylated peptide 41 on the proliferation of the SK-BR-3 breast cancer cells. **A**) Protein expression. The endogenous Grb7 protein expression (left) and expression of the fusion protein of 41-mRLUC-3×Flag (right) in the SK-BR-3 breast cancer cells were identified by western blot. **B**) SK-BR-3 breast cancer cell proliferation analysis. Double-factor variance analysis was performed to analyze the differences between the adjusted absorbance and different transfected cell groups, and between the adjusted absorbance and different time points. Statistically significant differences existed in both of groups (n = 10, p<0.001).

The effect of nonphosphorylated peptide 41 on the proliferation of SK-BR-3 breast cancer cells, which express high levels of the Grb7 protein [32], was evaluated using the MTS (3-(4,5-dimethylthiazol-2-yl)-5-(3-carboxymethoxyphenyl)-2-(4- sulfophenyl)-2H-tetrazolium, inner salt) cell proliferation assay. Endogenous expression of the Grb7 protein and expression of the fusion protein of 41-mRLUC-3×Flag in SK-BR-3 breast cancer cells were first verified (Figure 4A). Beginning from 24 h, the absorbance of the 41-mRLUC-3×Flag group indicated that the proliferation of 41-mRLUC-3×Flag-transfected SK-BR-3 breast cancer cells was significantly slower than that of the control group (p<0.01) (Figure 4B).

## Discussion

In this study, we identified a novel nonphosphorylated motif that bound to the SH2 domain of Grb7 protein with an affinity at least comparable to the phosphorylated ligand pY (1180). This twenty-two amino acid-motif contains conserved amino acids at the N- and C-terminus and fifteen amino residues between that are enriched with proline (21%), threosine (18%) and serosine (13%) (Table 1). The binding affinity of the nonphosphorylated n–Y–c peptide to the SAP SH2 domain is also similar to that of phosphorylated ligands [33]. To our knowledge, this is the first report to highlighting a protein domain that recognizes such an extensive sequence motif structure (http://pawsonlab.mshri.on.ca/index.php).

SH2 domains promote rapid and reversible signaling transduction [34]. If a nonphosphorylated peptide has a higher affinity to the SH2 domain comparable to phosphorylated ligand, the interaction becomes independent of phosphorylation and signaling may not be rapidly attenuated [33]. The nonphosphorylated G7-18 NATE peptide [27] has been used to inhibit Grb7 protein functions. Mice injected with pancreatic cancer cells and treated with the G7-18NATE peptide have fewer peritoneal metastases compared to controls [3]. Inhibition of the G7-18NATE peptide on the proliferation of several breast cancer cell lines, including MDA-MB-231, ZR-75-30, SK-BR-3 and MDA-MB-361, is also significant [29]. In our study, the proliferation of SK-BR-3 breast cancer cells transfected with the 41-mRLUC-3×Flag plasmid was significantly slower than that of the mRLUC-3×Flag control group (Figure 4B).

We showed that the peptide 41 was more stable than peptide 10 (Figure 1A), possibly due to the presence of two tandem motifs in peptide 41. Peptide 10 showed a slightly stronger BRET signals compared to peptide 41 (Figure 2B). These nonphosphorylated peptides may be optimized and useful for the rational design of drugs targeted against cancers that harbor increased Grb7 protein expression.

## Materials and Methods

### Materials

The ABL_1 and Grb2 plasmids were gifts from Professor Ruibao Ren (Brandeis University, USA), and the Grb7 (BC006535) and Grb14 cDNA (BC053559) plasmids were from the Proteintech Group. The inserted sequences of the SH2 domains were as follows: **Grb7**: N-IHRTQLWFHGRISREESQRLIGQQGLVDGLFLVRESQRNPQGFVLSLCHLQKVKHYLILPSEEEGRLYFSMDDGQTRFTDLLQLVEFHQLNRGILPCLLRHCCTRVAL-C; **Grb2**: N-EMKPHPWFFGKIPRAKAEEMLSKQRHDGAFLIRESESAPGDFSLSVKFGNDVQHFKVLRDGAGKYFLWVVKFNSLNELVDYHRSTSVSRNQQIFLRDIEQVPQQP-C; **ABL_1**: N-SLEKHSWYHGPVSRNAAEYLLSSGINGSFLVRESESSPGQRSISLRYEGRVYHYRINTASDGKLYVSSESRFNTLAELVHHHSTVADGLITTLHYPAPKRNK-C; and **Grb14**: N-QSSATNMAIHRSQPWFHHKISRDEAQRLIIQQGLVDGVFLVRDSQSNPKTFVLSMSHGQKIKHFQIIPVEDDGEMFHTLDDGHTRFTDLIQLVEFYQLNKGVLPCKLKHYCARIAL-C.

In the mutation assay, the control and mutation sequences were as follows:

10: N-AGVESLGIPNYTPTT PTLLLTRPLPGIPRGLRGW-C;

10AM: N-AGVESL YTPTTPTLLLTRPLPGIPRGLRGW-C;

10BM: N-AGVESLGIPNYTPTTPTLLLTRPLP RGLRGW-C;

and 10CM: N-AGVESL YTPTTPTLLLTRPLP RGLRGW-C.

In the co-immunoprecipitation assay, the mRLUC fragment was generated by PCR amplification. The inserted sequence of the Grb7 SH2 domain was as follows: N- IHRTQLWFHGRISREESQRLIGQQGLVDGLFLVRESQRNPQGFVLSLCHLQKVKHYLILPSEEEGRLYFSMDDGQTRFTDLLQLVEFHQLNRGILPCLLRHCCTRVAL-C. This sequence was inserted at the C terminus of the EGFP tag. The sequence of peptide 10 was as follows: N-GMWEGLEGGGVAGVESLGIPNYTPTTPTLLLTRPLPGIPRGLRGWSLLRCCRLLCRNSYITAAPKHHHPLQTSPRGSPGLQEFDIKLIDTVDLEGGPGTQFAL-C. The sequence of peptide 41 was as follows: N-VGTWTTRGPWCPCVAVGIPTQPTTSSEPSPPSNPPWDPGRVLLGRIVWPGLLALGIPTHHQNDTYNSPHAHPNRDP-C. The sequence of peptide 10CM was as follows: N-AGVESLYTPTTPTLLLTRPLPRGLRGW-C. The nonphosphorylated peptide and the mRLUC fragment were inserted at the N-terminus of the Flag tag. The mRLUC fragment was fused with the nonphosphorylated peptide so that they could be easily identifiable. The empty vector containing the mRLUC fragment was used as the negative control.

The phosphorylated ligand pY (1180) (DEEYEpY(1180)MNRRR) from the ErbB3 and the nonphosphorylated peptide 41-A (VAVGIPTQPTTSSEPSPPSNPPWDPG RV) from nonphosphorylated peptide 41 were synthesized by the China Peptides Co., Ltd. The HPLC purification analysis and MS quality analysis reports were obtained.

For the BRET assay, the EYFP fragment was generated by PCR amplification. The ErbB3-mRLUC plasmid was constructed by the Beijing Sino Biological Inc. The SH2 domain of Grb7 was fused to the EYFP fragment, and the nonphosphorylated peptides 10 and 41 were fused to the mRLUC fragment. The Grb7 SH2 domain sequence was inserted at the C-terminus of the EYFP tag.

For the cell proliferation assay, the anti-Grb7 antibody (N-20, Santa Cruz Biotechnology, USA) and the anti-Flag antibody (M20008M, Abmart, China) were used for the western blot, and MTS (Promega, USA) was used for the analysis of cell proliferation.

### Methods

#### Yeast two-hybrid system

The GAL4 BD-SH2 fusion bait plasmids were transformed into the yeast strain CG1945 using the lithium acetate protocol. The transformants were grown on SD/-Trp plates and spread on SD/-Trp-His plates for self-activation estimation. Those transformants without background growth or growth inhibited by 3-amino-1, 2, 4-triazole were selected for the subsequent screening. Approximately 3.01×10^6^ clones of the random peptide library (Protocol No. PT303921, Clontech, USA), as AD baits, were effectively transformed into yeast strain CG 1945 containing BD bait plasmids and used to be screened according to the MATCHMAKER Two-Hybrid System protocol (Cat. No. 630489, Clontech, USA). Plasmids of potential positive transformants selected on plates with SD/-Trp-His-Leu medium in the primary screening were isolated and retransformed into the CG1945 cells containing the corresponding SH2 bait plasmids. The improved LacZ assay was then performed. Clones that were positive for all of the reporter assays and confirmed by three independent improved LacZ tests were selected for specific interactions [35].

#### Cell transient transfection

HEK293T cells [36] at 70%–80% confluency were given fresh DMEM (high glucose) culture medium and transfected with plasmids using the calcium phosphate method. Samples were collected at 36 h after transfection.

#### Co-immunoprecipitation assay and western blot analysis

HEK293T cells were washed 5 times with ice-cold PBS buffer and scraped off the plates into cell lysis buffer (1% Nonidet P-40, 0.02 M TRIS (pH 7.5), 0.15 M sodium chloride, 0.001 M EDTA, 0.5% sodium deoxycholate, 0.1% SDS and 0.001 M PMSF). Lysates were incubated on ice for 5 minutes, vortexed 2 times for 30 seconds each and centrifuged at 12,000 rpm for 15 minutes at 4°C. After centrifuging twice, the supernatant was incubated with anti-Flag antibody (1∶100, M20008M, Abmart, China) for 6–8 h at 4°C. Protein G agarose (40 µL) was added into 1 mL of the above mixture and coupled to the anti-Flag antibody for 3 h at 4°C. The immunoprecipitates were washed 2–3 times with PBS buffer (PH 7.4), and the sediments were diluted with 5×SDS electrophoresis buffer and heated at 97°C for 10 minutes. The proteins were separated by SDS-PAGE and transferred to PVDF membranes (IPVH00010, Millipore, USA). The membranes were incubated with anti-GFP antibody (1∶2000, P30010, Abmart, China) and goat anti-rabbit secondary antibody (1∶4000, M21002, Abmart, China) to identify target proteins. For the competition analyses, the immunoprecipitation complexes were mixed with anti-Flag antibody and different concentrations of the synthesized phosphorylated ligands or the nonphosphorylated ligands. The proteins were separated by SDS-PAGE, and the SH2 domain of Grb7 protein was analyzed using anti-GFP antibody. Western blot analysis was carried out using the Image J [37], the IC_50_ values were calculated by regression analyses using SPSS 17.0.

#### Bioluminescence Resonance Energy Transfer (BRET)

In BRET, the Renilla luciferase (mRLUC) is fused to the candidate protein, and the green fluorescent protein mutant (EYFP) is fused to the target protein. Interactions between the two fused proteins bring the luciferase and the green fluorescent protein into close proximity, which promotes resonance energy transfer from mRLUC to EYFP [38]. Then EYFP is excited at 530 nm, and the emission is used to determine the binding between the two interacting proteins. In this study, HEK293T cells were collected at 36 h after transfection, 100 µL of mixed cell suspension was added to the u-bottom 96-well microtiter plates (Corning, USA) before measurement. The measurement of bioluminescence, with rasters of 460/40 nm and 518/20 nm, was performed on Synergy4 Multi-Mode Microplates Readers (BioTek, USA). The BRET reaction was initiated by 5.9 µM coelenterazine (Promega, USA). Data were analyzed by one-factor analysis of variance using SPSS 17.0.

#### Structural model of Grb7 SH2 domain binding to nonphosphorylated peptide

The structure of the nonphosphorylated ligand 41-A (GIPTQPTTSSEPSPPSNPP WDP) was first built by MOE. This sequence had no homology sequence with known structure in the Protein Date Bank (PDB) database and no predicted secondary structure, such as α helix or β strand element. Therefore, a linear, extended conformation was set as the primary conformation of this ligand. Then, the energy of this ligand was minimized in an AMBER99 force field to build its final conformation for docking. Then the structure of the human Grb7 SH2 domain complex with a ten amino acid-peptide was downloaded from the PDB database (ID: 2l4k). The structure of the SH2 domain was extracted for docking. Thus, a docking method was used to predict the structure of the domain-ligand complex.

#### Cell proliferation assay

The MTS cell proliferation assay was performed to analyze the proliferation of SK-BR-3 breast cancer cells (Cell culture center of PUMC, China) according to the manufacturer's instructions (Promega, USA). SK-BR-3 cells at 70%–80% confluency were transiently transfected respectively with plasmids of 41-mRLUC-3×Flag or mRLUC-3×Flag and Mega Tran 1.0 (OriGene, USA). Beginning from 12 h after transfection, 400 µg/mL G418 (11811-023, Invitrogen, USA) was used to select for the transfected cells [39]. The u-bottom 96-well microtiter plates (Corning, USA) were seeded with approximately 7.50×10^3^ cells per well at 18 h after transfection. 20 µL of MTS was added into 100 µL of cell culture medium, and the mixtures were incubated at 37°C for 1 h before measurement. The absorbance at 492 nm of transfected cells and control DMEM medium was measured separately at 18 h, 24 h, 36 h, 48 h and 60 h after transfection on Synergy4 Multi-Mode Microplates Readers (BioTek, USA). The absorbance was adjusted by differences in the absorbance between cell culture medium and control medium without seeded cells. The data were analyzed by double-factor variance analysis using SPSS 17.0.

## Supporting Information

Table S1 Amino acid sequences of nine nonphosphorylated peptides.
(DOC)
