# Supplementary material for: Novel Nonphosphorylated Peptides with Conserved Sequences Selectively Bind to Grb7 SH2 Domain with Affinity Comparable to Its Phosphorylated Ligand
Source: PLoS One. 2012 Jan 11;7(1):e29902. doi: 10.1371/journal.pone.0029902 (PMC3256188; doi:10.1371/journal.pone.0029902)
Supplement: Table S1 — Amino acid sequences of nine nonphosphorylated peptides. (DOC) [file pone.0029902.s001.doc]

**Table S1 Amino acid sequences of nine nonphosphorylated peptides**

| **No. of peptide** | **Sequence** |
| --- | --- |
| 3 and 52 | N-IWSVGCRVEGMRCLGRNSYFEHTHKSTLRPIHTGIPR  RFGFASGESVFAVVL**GIPTHSSPQYSPPSTYSPPGDP**-C |
| 10 | N-GMWEGLEGGGVAGVESL**GIPNYTPTTPTLLLTRPLPG**  **IP**RGLRGWSLLRCCRLLCRNSYITAAPKHHHPLQTSPRG  SPGLQEFDIKLIDTVDLEGGPGTQFAL-C |
| 16 | N-WARIIGVVGACVAVVV**GIPTATTSPYENANPPHQTWD**  **P**-C |
| 41 | N-VGTWTTRGPWCPCVAV**GIPTQPTTSSEPSPPSNPPWD**  **P**GRVLLGRIVWPGLLAL**GIPTHHQNDTYNSPHAHPNRDP**  -C |
| 60 | N-PRYTEKVVRRRGLMVL**RNSYFTFLPARSLYLIKTHWD**  **P**-C |
| 67 | N-ITCQVIWRGVLGGCWCRNSYDPPLYRRPASYVYVPAG  SLGFAVAEGADGDFFEVV**GIPKAQNTTATPEQHASPTGI**  **P**-C |
| 98 | N-GEREMCWSRGERRFVIRNSYRLTLPVQSTIRFLSNMG  SLWYYVAGDVESWALGEV**GIPNQDPPAATQSPSQETTWD**  **P**-C |
| 106 | N-LTYFRGNAVVQPGGLWV**GIPTSTPNTHSTTSHHKNPW**  **DP**DSGRVVGGRFILCVGVR-C |
